# Plasmapheresis and Cyclophosphamide therapy in an eight year old girl with cerebral manifestation of systemic lupus erythematodes

**DOI:** 10.1186/1546-0096-9-S1-P259

**Published:** 2011-09-14

**Authors:** A Ulbrich, I Lanator, D Csaicsich, MT Schmook, R Seidl, W Emminger

**Affiliations:** 1 General Hospital of Vienna, Austria, Department of Pediatrics and Adolescent Medicin; 2 General Hospital of Vienna, Austria, Department of Radiology, Division of Neuro- and Musculoskeletal Radiology

## 

An eight year old girl suffering from systemic lupus erythematodes (SLE) presented with fever and developed a slurred and slowed speech, followed by hemiparesis of the right side, tremor of the arms and an instable gait. Diagnosis of SLE was done two years ago during an episode of muscle pain and polyarthralgia. From December 2008 until August 2009 the girl has received corticosteroids, naproxen and methotrexate.

The cerebral magnetic resonance imaging (MRI) showed multiple T2-weighted hyperintense lesions in the cerebellum, periventricular on the right hemisphere, putamen bilateral and white matter of the frontal lobe. The blood results revealed a consumption of the complement system with decreased values of C3, C4 and CH50, increases of serum inflammatory factors, high values of antinuclear antibodies and of antibodies against dsDNA. Antibodies against SSA (Ro), SSB (La), u1RNP and SM have also been positive for a long time. Antibodies against phospholipids were not detected.

High doses of corticosteroids (30 mg/kg/day methylprednisone) have been applied for three days and were followed by an apparent improvement of the hemiparesis. Speech worsened and an organic psychosyndrome developed with confusion, aggressiveness and a loss of short term memory and orientation. Plasmapheresis therapy on three consecutive days was performed and was followed by a prompt improvement of the organic psychosyndrome.

After the second plasmapheresis procedure the girl developed an insult with a seizure. An MRI showed new hyperintense T2 lesions in the occipito-parieto-temporal area and in the frontal lobe in the white matter without restricted diffusion. Most of the known former lesions showed a dramatic improvement.

Nine days after plasmapheresis and seven days after the first cyclophosphamide therapy the patient showed an impressive clinical improvement. Four weeks after the first symptoms the hyperintense lesions were in regression and the complement levels were within normal limits. The immunosuppressive therapy consisted of daily corticosteroids and intravenous cyclophosphamide in four weekly intervals and a continuous anticoagulation has been applied.

Now ten months after the severe cerebral manifestation of SLE, the girl is well and going to school without any neurological handicap.

